# Olfactory context and memory across the adult lifespan: effects on mnemonic discrimination and olfactory function

**DOI:** 10.3389/fnagi.2026.1917760

**Published:** 2026-09-28

**Authors:** Renae Mannion, Alexis Bailey, Stephanie L. Leal

**Affiliations:** 1 Department of Psychological Sciences, Rice University, Houston, TX, United States; 2 Department of Integrative Biology & Physiology, University of California, Los Angeles (UCLA), Los Angeles, CA, United States

**Keywords:** aging, hippocampus, memory, mnemonic discrimination, odor, olfaction

## Abstract

Odor is a powerful contextual cue that can influence memory and is closely linked to brain regions involved in episodic memory, including the hippocampus. Age-related olfactory dysfunction is among the earliest behavioral changes associated with cognitive decline and may contribute to memory impairments in older adults. Hippocampal pattern separation, or the process of reducing interference among similar experiences, provides a sensitive measure of age-related memory decline, yet little is known about how olfactory context influences mnemonic discrimination. In the present study, we examined whether odor context during encoding affects subsequent mnemonic discrimination in young and older adults. Participants completed a mnemonic similarity task in which object stimuli were encoded in both odor (lavender) and no-odor contexts prior to a memory test. Across participants, odor context did not significantly influence target recognition or mnemonic discrimination. Consistent with prior work, older adults exhibited poorer mnemonic discrimination and reduced olfactory function relative to young adults. However, odor presentation order influenced mnemonic discrimination in older adults, such that performance was better when the odor context was encountered during the first encoding block compared to the second. Additionally, poorer olfactory function was associated with reduced mnemonic discrimination, and this relationship was largely explained by age-related differences in olfactory ability. These findings suggest that the influence of olfactory context on memory depends on both age and the timing of odor exposure, highlighting the importance of individual differences in olfactory function when evaluating odor-based approaches to support memory in aging.

## 1 Introduction

Olfaction, or the sense of smell, is uniquely intertwined with memory, exhibiting a potent ability to evoke vivid, emotional memories. Unlike other sensory modalities, olfactory input bypasses the thalamus and directly engages brain regions such as the amygdala and hippocampus, which are critical for emotional and memory processing (Herz and Engen, 1996; Savic, 2002; Sullivan et al., 2015). This privileged neuroanatomical access may underlie the strong contextual and emotional associations linked to odor-evoked memories. Understanding how olfactory cues influence memory processing may provide insight into both fundamental cognitive mechanisms and age-related changes in memory function (de Moraes e Silva et al., 2018).

Olfactory dysfunction is a common but often overlooked consequence of aging, affecting nearly half of older adults to varying degrees (Doty and Kamath, 2014). Unlike other sensory declines, loss of smell can have profound implications for health and cognition, including reduced quality of life, impaired nutrition, and diminished safety awareness (Croy et al., 2014). Importantly, olfactory impairment has been linked to age-related cognitive decline and neurodegenerative disease (Devanand et al., 2015). The olfactory system is uniquely vulnerable to aging because of its direct exposure to environmental insults and its strong anatomical and functional connections with memory-related brain regions, including the hippocampus and entorhinal cortex (Attems et al., 2015; Murphy, 2019). Notably, both the olfactory bulb and dentate gyrus (DG), a hippocampal subfield critical for pattern separation (Yassa and Stark, 2011; Leal and Yassa, 2018), are among the few brain regions associated with adult neurogenesis (Sahay et al., 2011) and exhibit age-related changes that may contribute to declines in olfactory processing and memory. Understanding how olfactory function relates to hippocampal-dependent memory may therefore provide insight into shared mechanisms underlying cognitive aging.

Alongside olfactory dysfunction, aging is also associated with declines in episodic memory, particularly in the ability to distinguish between similar experiences, a process known as hippocampal pattern separation (Yassa and Stark, 2011). This computation is critically supported by the dentate gyrus (DG) region of the hippocampus, which transforms overlapping inputs into distinct, non-overlapping representations (Treves and Rolls, 1994). Age-related dysfunction of hippocampal circuits, especially in the DG and CA3 subfields, has been linked to impairments in this process, resulting in increased memory interference and reduced mnemonic precision (Holden et al., 2013). To assess pattern separation in humans, mnemonic similarity tasks (MST) have been developed which require individuals to differentiate between previously seen items, similar lures, and entirely new stimuli, thereby taxing hippocampal pattern separation (Stark et al., 2013). Performance on these tasks has been shown to decline with age and is associated with neurobiological markers of hippocampal integrity, making them a sensitive behavioral tool for detecting early cognitive changes (Stark et al., 2013; Yassa et al., 2011). Thus, hippocampal pattern separation deficits represent a promising target for understanding and tracking memory dysfunction in the aging brain (Leal and Yassa, 2018).

Emerging evidence suggests that olfactory cues may play a unique role in hippocampal-dependent memory processes, including pattern separation. Theoretical and experimental work has proposed that neurogenesis within both the olfactory bulb and dentate gyrus may contribute to the formation of distinct representations for overlapping sensory experiences, thereby supporting discrimination of similar odors, contexts, and events (Sahay et al., 2011). Given the strong anatomical connectivity between olfactory pathways and medial temporal lobe structures (Zhou et al., 2021), odors may serve as salient contextual features during memory encoding and retrieval. Despite age-related declines in olfactory function, recent studies suggest that odor-based interventions may support cognition in older adults, with olfactory enrichment paradigms producing improvements in memory and neural functioning (Chen et al., 2021; Oleszkiewicz et al., 2018; Woo et al., 2023). These findings raise the possibility that olfactory context may influence hippocampal-dependent memory processes in aging.

Behavioral evidence further supports a close relationship between olfactory function and memory across aging. Older adults with poorer odor identification tend to exhibit poorer episodic memory, and longitudinal studies suggest that changes in olfactory function and episodic memory can track one another over time (Knight et al., 2020; Dintica et al., 2021). In a large population-based sample of older adults, olfactory identification was associated with episodic memory and differentiated patterns of cognitive decline across aging (Dintica et al., 2021). Similarly, olfactory memory itself declines with advancing age, with age-related differences particularly evident in older and very old adults (Larsson et al., 2016). A recent meta-analysis further demonstrated significant associations between olfactory function and declarative memory in cognitively normal older adults (Jobin et al., 2023). Together, these behavioral findings suggest that olfactory and memory systems may be closely linked during aging, although the mechanisms underlying this relationship remain unclear.

It is important to note that odor perception and its influence on responses can vary depending on the stimulus itself (i.e., intensity and frequency of exposure), the individual perceiver (i.e., sex, age, emotional state), and the context surrounding the exposure (i.e., other senses co-stimulated, semantic knowledge, and cultural background) (Rouby et al., 2009). Given the intimate connection between the olfactory system and brain regions associated with emotion, the emotional valence of smells, or whether they are perceived as pleasant or unpleasant, can have significant effects on both behavioral and cognitive processes. Importantly, substantial individual differences exist in odor perception, including perceived pleasantness, familiarity, and intensity, all of which may influence how odors affect cognition and memory (Herz, 2016). These individual differences may be particularly relevant in older adulthood, where variability in olfactory function increases with age.

Importantly, behavioral studies cannot determine whether olfactory cues directly alter hippocampal computations involved in mnemonic discrimination. Neuroimaging approaches may therefore help determine whether odor exposure during encoding modulates activity or connectivity within hippocampal and olfactory memory networks. The present study provides a behavioral foundation for this question by examining whether a brief olfactory context influences mnemonic discrimination and whether this effect differs across age.

In the current study, we examined whether odor context (lavender presented through a diffuser) during encoding influences subsequent object memory and mnemonic discrimination relative to a within-subject no-odor encoding condition. Because both olfactory function and hippocampal-dependent memory decline with age, we included young and older adults to determine whether age moderates the influence of odor context on memory. We hypothesized that odor context would enhance memory performance, particularly mnemonic discrimination, given the strong anatomical and functional links between olfactory and hippocampal memory systems, relative to a no-odor context. Given evidence for substantial individual differences in odor perception and olfactory function, we also explored whether age, olfactory function, odor presentation order, and subjective odor characteristics were associated with memory performance.

## 2 Materials and methods

### 2.1 Participants

Participants ages 18–35 (*N* = 25) and 60–85 (*N* = 25) were recruited from Rice University and the local Houston community through flyers and listserv postings. Participants were compensated with either cash or an Amazon gift card ($15/hour) upon completion of the study. Informed consent was obtained from all participants in accordance with the guidelines approved by the Rice University Institutional Review Board. This study complies with the Declaration of Helsinki for research involving human subjects. All participants had no major medical, psychiatric, or substance abuse history and had normal or corrected-to-normal vision. Participants were instructed not to wear personal fragrance (e.g., lotion, perfume, or sunscreen) or eat or drink anything besides water an hour prior to their sessions. Data from two older adults and one young adult were excluded due to technical issues or task response errors that precluded calculation of memory performance measures. The final sample consisted of 24 young adults and 23 older adults. Participant demographics are shown in Table 1.

**TABLE 1:** Participant demographic information.

	Overall		Group 1		Group 2	
Variable (mean ± SD)	Young	Old	Young	Old	Young	Old
*N*	24	23	13	12	11	11
Age, *p < 0.001, *d* = −8.022	24.13 (5.15)	67.22 (5.59)	23.69 (4.84)	66.25 (5.56)	24.64 (5.70)	68.27 (5.69)
Sex, ∼*p* = 0.065, *d* = −0.549	13M, 11F	5M, 18F	7M, 6F	5M, 7F	6M, 5F	0M, 11F
Education (years), *p = 0.017, *d* = −0.723	15.42 (2.47)	17 (1.86)	14.92 (2.06)	16.92 (1.68)	16 (2.86)	17.09 (2.12)
Race/ethnicity (%), *p = 0.023, *d* = 0.685						
White	31%	83%	54%	75%	18%	91%
Black/African American	7%	17%	8%	25%	9%	9%
Asian/Asian American	34%	0%	31%	0%	55%	0%
Other	28%	0%	8%	0%	18%	0%
Hispanic/Latino/a/x	29%	0%	31%	0%	27%	0%

*Significant overall group differences (young vs. old) *p* < 0.05, ∼ = marginal *p* < 0.07.

### 2.2 Questionnaires

Participants completed a demographics form to determine age, sex, race, and ethnicity. Participants also completed two series of questionnaires during the study. The first series of questionnaires were administered between encoding and retrieval. Participants completed the Beck Depression Inventory-II (BDI-II; Beck et al., 1996) to assess current depressive symptoms, which may influence both olfactory and memory performance (Dohm-Hansen and Johansson, 2020; Camfield et al., 2018), olfactory function (Kim and Bae, 2025), and odor memory (Kazour et al., 2020; Naudin et al., 2014). Participants also completed a Lifestyle and Exercise Questionnaire (LEQ), which we developed to measure healthy habits such as physical and social activity, mindfulness, and eating a well-balanced diet, an Allergies Questionnaire which we developed to measure allergy triggers and symptoms that could impact olfaction, and a Smoking Questionnaire which we developed to measure experience with using nicotine products that could impact olfaction.

The second set of questionnaires was administered after the retrieval phase. Participants completed a modified version of the Odorant Rating Scale (Moss et al., 2016) to measure perception of the test odor (i.e., pleasantness, intensity, complexity, etc.) and ability to identify the test odor, the Odor Awareness Scale (Smeets et al., 2008) to measure individual differences in awareness of odors in the environment, and the Subjective Memory Complaints Questionnaire (Youn et al., 2009) to measure subjective memory complaints for general memory and for everyday memory. Participants also completed the University of Pennsylvania Smell Identification Test (UPSIT; Doty et al., 1984) to assess olfactory function. Questionnaire results are listed in Table 2.

**TABLE 2:** Questionnaire results across young and older participants.

	Overall		Group 1		Group 2	
Variable (mean ± SD)	Young	Old	Young	Old	Young	Old
Beck Depression Inventory (BDI-II)	8.38 (6.72)	7.70 (7.03)	8.77 (6.42)	8.08 (6.04)	7.91 (7.34)	7.27 (8.26)
Lifestyle and Exercise Questionnaire (LEQ)						
General health score	3.25 (0.94)	3.70 (0.76)	3.46 (1.05)	3.75 (0.62)	3.00 (0.77)	3.64 (0.92)
Allergies Questionnaire						
Do you suffer from allergies?	1.54 (0.51)	1.45 (0.49)	1.54 (0.52)	1.42 (0.51)	1.55 (0.52)	1.27 (0.47)
Smoking Questionnaire						
Ever used any nicotine product(s)? *p = 0.004, *d* = 0.892	1.83 (0.38)	1.43 (0.51)	1.77 (0.44)	1.42 (0.51)	1.91 (0.30)	1.45 (0.52)
Odorant Rating Scale (ORS)						
Total score	38.83 (6.91)	36.43 (6.63)	38.23 (6.27)	36.17 (7.65)	39.55 (7.85)	36.73 (5.68)
Familiarity score	6.54 (1.91)	6.09 (1.16)	6.54 (1.76)	5.92 (1.16)	6.55 (2.16)	6.27 (1.19)
Intensity score, *p = 0.008	4.25 (1.82)	2.83 (1.70)	3.62 (1.33)	2.33 (1.61)	5.00 (2.10)	3.36 (1.69)
Pleasantness score	7.54 (1.25)	7.39 (1.47)	7.08 (1.38)	7.33 (1.67)	8.09 (0.83)	7.45 (1.29)
Irritation score	1.83 (1.34)	1.83 (1.61)	1.69 (0.85)	2.00 (1.95)	2.00 (1.79)	1.64 (1.21)
Frequency of encounter score	4.04 (1.94)	4.57 (1.59)	4.46 (1.94)	4.33 (1.78)	3.55 (1.92)	4.82 (1.40)
Complexity score	4.79 (1.82)	4.87 (2.03)	5.15 (1.52)	4.83 (2.17)	4.36 (2.11)	4.91 (1.97)
Ease of description score	4.38 (2.02)	3.61 (1.99)	4.38 (2.10)	4.00 (2.41)	4.36 (2.01)	3.18 (1.40)
Ease of imagining score	5.46 (2.52)	5.26 (2.12)	5.31 (2.29)	5.42 (2.43)	5.64 (2.87)	5.09 (1.81)
Odor Awareness Scale (OAS)						
Total score	103.50 (19.06)	109.61 (15.04)	103.38 (19.89)	103.92 (15.86)	103.64 (18.99)	115.82 (11.81)
Positive score	38.58 (7.15)	42.13 (6.08)	38.85 (7.50)	40.50 (6.80)	38.27 (7.06)	43.91 (4.87)
Negative score	64.92 (12.31)	67.48 (10.44)	64.54 (12.51)	63.42 (10.87)	65.36 (12.67)	71.91 (8.25)
SMCQ	2.63 (2.60)	2.22 (1.93)	3.54 (3.10)	2.33 (1.67)	1.55 (1.29)	2.09 (2.26)
UPSIT, *p = 0.020, *d* = 0.704	33.08 (2.75)	30.09 (5.40)	33.85 (2.70)	28.92 (6.71)	32.18 (2.64)	31.36 (3.35)

Values are Mean (Standard Deviation); BDI = scores range from 0 to 63 points; LEQ = General health scores range from 1 to 5, where higher scores indicate greater reported levels of general health; Allergies Questionnaire = scores range from 1 to 2, where scores 1 = Yes and 2 = No; Smoking Questionnaire = scores range from 1 to 2, where 1 = Yes and 2 = No; ORS = Total scores range from 8 to 72 points, where higher scores indicate greater agreement across dimensions (i.e., “strongly agree” the odor is familiar, intense, etc.) and lower scores indicate lesser agreement (i.e., “strongly disagree”); Individual item scores (i.e., pleasantness, complexity, etc.) range from 1 to 9, where higher scores indicate greater perceived pleasantness, complexity, etc. of the test odor; OAS = Total scores range from 31 to 154 points, where higher sum scores indicate greater awareness of environmental odors; Positive scores range from 11 to 55, where greater scores indicate greater awareness of positive odors; Negative scores range from 20 to 100, where greater scores indicate greater awareness of negative odors; Subjective Memory Complaints Questionnaire (SMCQ) = scores range from 0 to 14 points, where higher scores indicate more severe memory complaints; University of Pennsylvania Smell Identification Test (UPSIT) = scores range from 0 to 40 points, where lower scores indicate worse olfactory function; *Significant overall group difference (Young vs. Old), *p* < 0.05.

### 2.3 Mnemonic similarity task

The MST is a well-established object recognition memory task that is thought to tax hippocampal pattern separation (Stark et al., 2013). The traditional version of the MST consists of an incidental encoding phase and a retrieval test phase. During the encoding phase, participants made indoor/outdoor judgements for 128 images (3s each, 1s ISI, color objects on a white background) via button press. To examine the effect of odor on memory encoding, the encoding phase of the MST was divided into two parts, taking place in separate testing rooms (see Supplementary Information 1 for room details) to avoid odor contamination across encoding blocks. Participants made indoor/outdoor judgements for 64 images in each room. In one room, participants were exposed to a test odor (lavender) using an essential oil diffuser during memory encoding. Participants completed the no-odor encoding block in a separate room. We counterbalanced the order that the scent was presented during encoding. Participants were randomly assigned to one of two counterbalancing orders: odor during Encoding Block 1 followed by no odor during Encoding Block 2, or no odor during Encoding Block 1 followed by odor during Encoding Block 2. This allowed us to examine the influence of odor and no-odor conditions on memory within-subjects (Figure 1).

**FIGURE 1:**
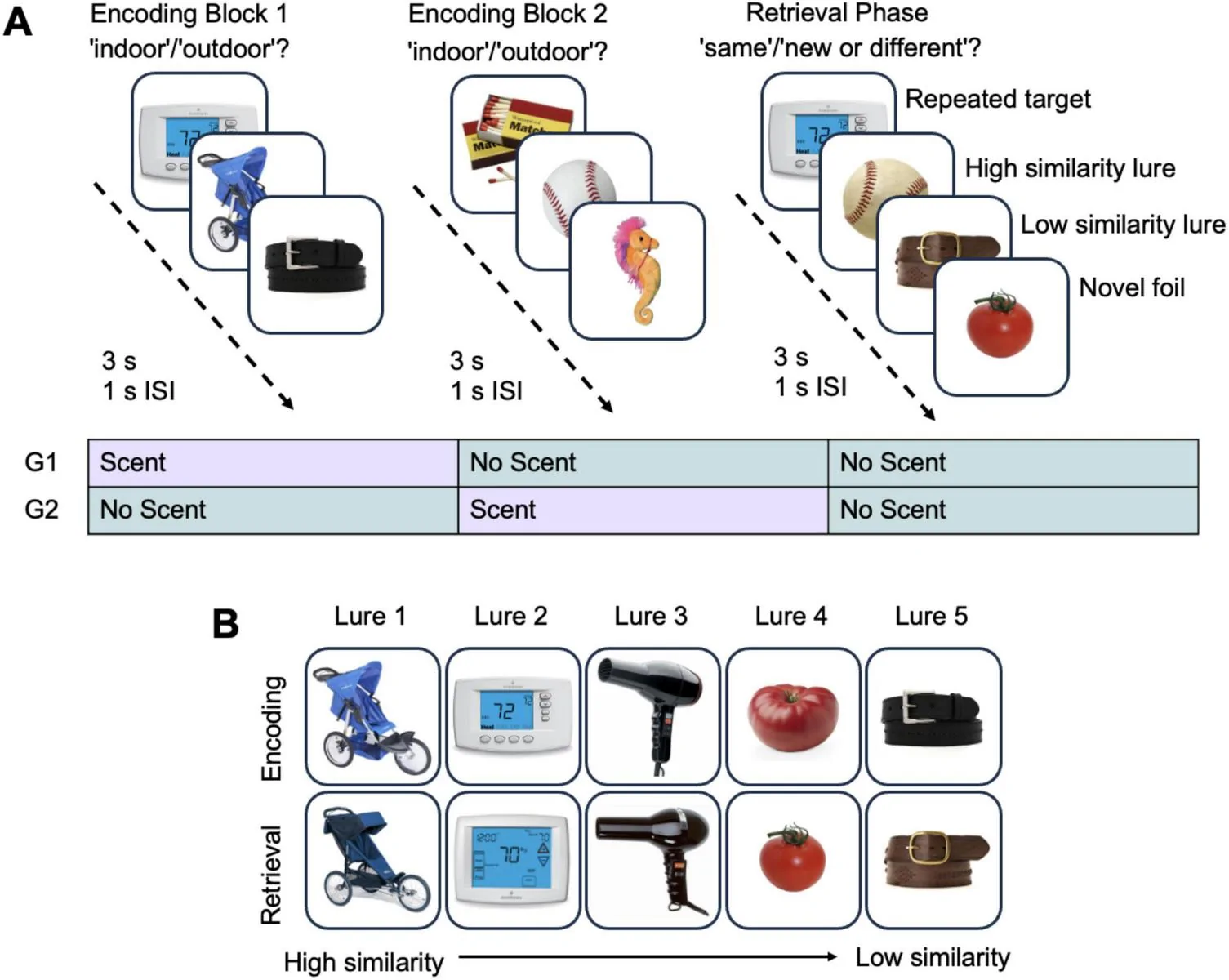
Mnemonic similarity task and study procedures across scent conditions. **(A)** Participants were shown a series of images during the Encoding phase and were asked to determine if each image was an “indoor” or “outdoor” item. After a short delay, participants were tested on their memory in the Retrieval phase and were shown either a repeated (target), new (foil), or a similar but not identical (lure) image. **(B)** Lure images varied by similarity level, such that high similarity lures were objects with less obvious differences (i.e., two blue baby strollers) whereas low similarity lures were objects with more obvious differences (i.e., two different colored belts). Images reproduced with permission from the Mnemonic Similarity Task (MST) dataset, Stark et al., 2013, https://github.com/celstark/MST.

The retrieval phase took place in the room without the odor after a series of questionnaires where participants were told to identify each item as “Same” or “New or Different” via button press. Here, participants responded to 192 images (3s each, 1s ISI). Images consisted of 64 exact repeats from both encoding phases (targets), 64 completely novel images (foils), and 64 images that were similar, but not identical to images seen during both encoding phases (lures). Additionally, the similar lures used varied in their degree of mnemonic similarity from very high similarity (L1) to very low similarity (L5). Images were selected from the open-source Mnemonic Similarity Task (MST) stimulus set developed by the Stark Laboratory (Stark et al., 2013), based on image resolution and relevance to modern, everyday life. The MST stimulus sets consist of color photographs of everyday objects and include matched target, lure, and foil stimuli spanning multiple levels of mnemonic similarity (Figure 1B). For details on exact breakdown of stimuli across conditions, see Supplementary Table 1.

### 2.4 Odor selection and administration

Lavender essential oil (*Lavandula angustifolia*) from Gya labs was chosen to be the experimental odor, as lavender has been reported to be the most widely purchased plant for essential oil extraction (Malloggi et al., 2022). Generally, lavender is viewed as a pleasant odor with relaxing properties, but there are individual differences in perceived odor pleasantness, which were examined in relation to memory performance (Degel and Köster, 1999; Moss et al., 2003; Nordin et al., 2017; Rouby et al., 2009). Lavender is also not an odor included in the UPSIT, which was administered to participants at the end of the study to assess their level of olfactory function. Lavender odor was delivered using an essential oil diffuser positioned behind the participant. This method minimized visual distraction, permitted standardized odor delivery across participants, and has been successfully employed in prior olfactory enrichment studies (Du et al., 2022; Woo et al., 2023).

Preliminary ratings were collected among six lab staff to determine the positioning of the diffuser relative to the participant, odor duration, and oil-to-water ratio. The diffuser was set up using 2/3 cup of water and three drops of essential oil and placed 75 in (190.5 cm) behind the participants as they sat at the testing computer. The diffuser was turned on during the consent form process and the participants entered the odor room between 10 and 20 min of the diffuser being turned on to ensure the odor intensity was consistent for each participant. For the odor to dissipate fully from the room, a minimum of 1 h with the door open was maintained between each participant being scheduled.

### 2.5 Memory measures of interest

We measured two key outcome measures: target recognition and lure discrimination. Target recognition (d’) is a standard memory measure of repeated items (targets), calculated as *d*’ = z(Hits) - z(False Alarms). Hits and false alarms refer to correct recognition (i.e., responding “same”) of repeated items and false recognition (i.e., responding “same”) of new items (foils), respectively. Lure discrimination (LDI) measures the ability to discriminate similar, but not identical items (lures) from targets, calculated as LDI = p(“New”| Lure) – p(“New”| Target) where responding “New” to a lure image is the correct response and corrected for response bias by subtracting responding “New” to repeated target images. Behavioral responses by trial type can be found in Supplementary Table 2.

### 2.6 Statistical analysis

Statistical analyses were conducted in SPSS v. 28 (IBM Corp., Armonk, NY). Primary analyses examined the effects of odor condition and age group on target recognition (d’) and lure discrimination (LDI) using mixed repeated-measures ANOVAs. Secondary analyses examined relationships between olfactory function (UPSIT) and memory performance using Pearson correlations and regression models. Given differences between age groups in sex distribution, race, and education, we conducted sensitivity analyses to examine whether the primary effects involving odor context, age, and odor presentation order were robust to the inclusion of these variables. Sex, race, and education were included as covariates in these analyses, and the pattern and statistical conclusions of the primary findings remained unchanged. Exploratory analyses examined whether odor presentation order and subjective odor characteristics were associated with memory performance using moderation, mediation, and stepwise regression approaches. Scheffé’s post-hoc contrasts and Pearson correlations were conducted where appropriate. Effect sizes are reported as η_*p*_^2^ or Cohen’s d where appropriate. Statistical values were considered significant at a final corrected alpha level of 0.05, which controlled for Type I error. Repeated-measures tests were corrected for error non-sphericity using Greenhouse-Geisser correction.

## 3 Results

### 3.1 No effect of scent condition on memory overall across age groups

The primary goal of this study was to examine whether scent used as a context during encoding of the MST may impact object memory compared to a within-subject no-odor encoding condition. Given changes in both odor perception and memory with age, we included a YA sample in addition to an OA sample to determine the impact of age on these effects. First, we conducted a mixed repeated measures ANOVA across scent condition (scent, no scent) as a within-subject factor and group (YA, OA) as a between-subject factor to examine memory for object stimuli. For target recognition, there were no significant effects for scent condition or age, or any interactions between scent condition and age (*p*’s > 0.05; Figure 2A). For lure discrimination, there was a significant effect of age [*F*(1,45) = 21.199, *p* < 0.001, η_*p*_^2^ = 0.320; Figure 2B], where YA showed better lure discrimination than OA. However, there were no significant effects for scent condition or any interactions between scent condition and age (*p*’s > 0.05).

**FIGURE 2:**
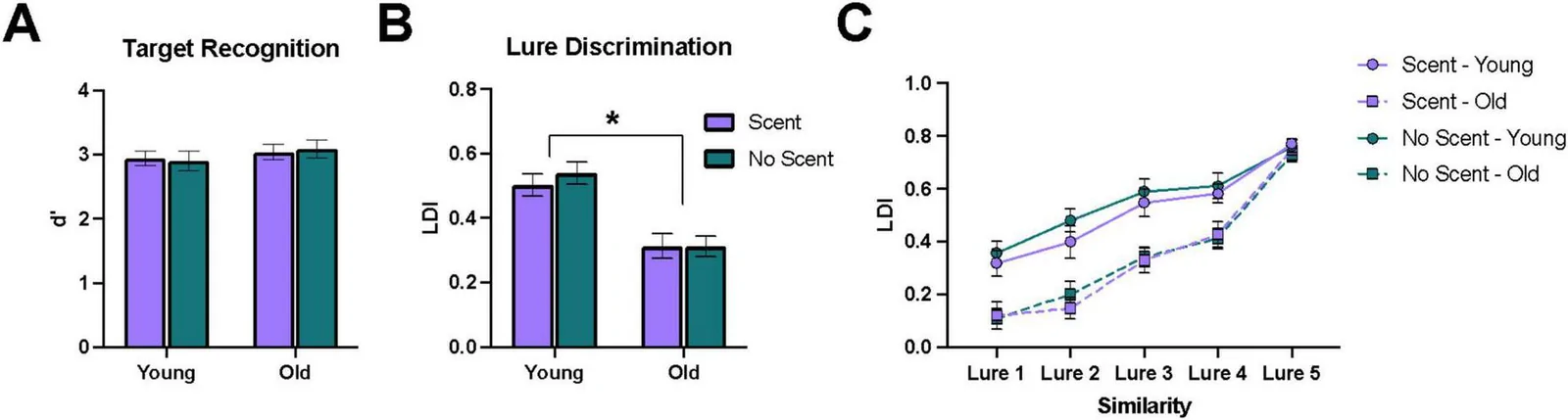
Mnemonic similarity task performance in young and older adults across scent conditions. **(A)** Target recognition (d’) performance in young and older adults across scent (lavender) and no scent (teal) during encoding conditions, **(B)** lure discrimination index (LDI) performance in young and older adults across scent (lavender) and no scent (teal) during encoding blocks, **(C)** LDI performance in young and older adults split by lure similarity bin from highest similarity (Lure 1) to lowest similarity (Lure 5). Error bars represent standard error of the mean (SEM). *Represents significant main effect of age group.

Next, we conducted a mixed repeated-measures ANOVA across scent condition (scent, no scent) and lure similarity bin (L1-L5) as within-subject factors and age (YA, OA) as a between-subject factor to examine memory across lure similarity. There was a significant main effect of similarity [*F*(4,180) = 152.877, *p* < 0.001, η_*p*_^2^ = 0.773; Figure 2C], where participants performed better with decreasing levels of similarity, and a main effect of age [*F*(1,45) = 19.704, *p* < 0.001, η_*p*_^2^ = 0.305], where YA performed better than OA, and a significant interaction between similarity and age [*F*(4,180) = 8.146, *p* < 0.001, η_*p*_^2^ = 0.153], exhibiting a linear relationship such that there were larger age-related differences in lure discrimination as lure similarity increased [*F*(1,45) = 24.115, *p* < 0.001, η_*p*_^2^ = 0.349]. However, there were no significant effects for scent condition or interactions between scent condition and age, scent condition and similarity, or scent condition, similarity, and age (*p*’s > 0.05). Thus, odor context did not significantly influence target recognition or mnemonic discrimination overall, whereas age-related differences in mnemonic discrimination were robust across analyses.

### 3.2 Age-specific effects of scent presentation order on memory performance

We counterbalanced the order in which scent was presented during encoding, in which Group 1 received the scent during Encoding Block 1 while Group 2 received the scent during Encoding Block 2 (see Figure 1). We conducted a mixed repeated-measures ANOVA across scent condition (scent, no scent) as a within-subject factor and age group (YA, OA) and scent condition order (G1 scent first, G2 scent second) as between-subject factors to examine potential order effects on memory for object stimuli. For target recognition, there were no significant effects for scent condition, order, age, or any interactions between these factors (all *p*’s > 0.05; Figures 3A, B). When we conducted these analyses within each age group, results remained the same (all *p*’s > 0.05).

**FIGURE 3:**
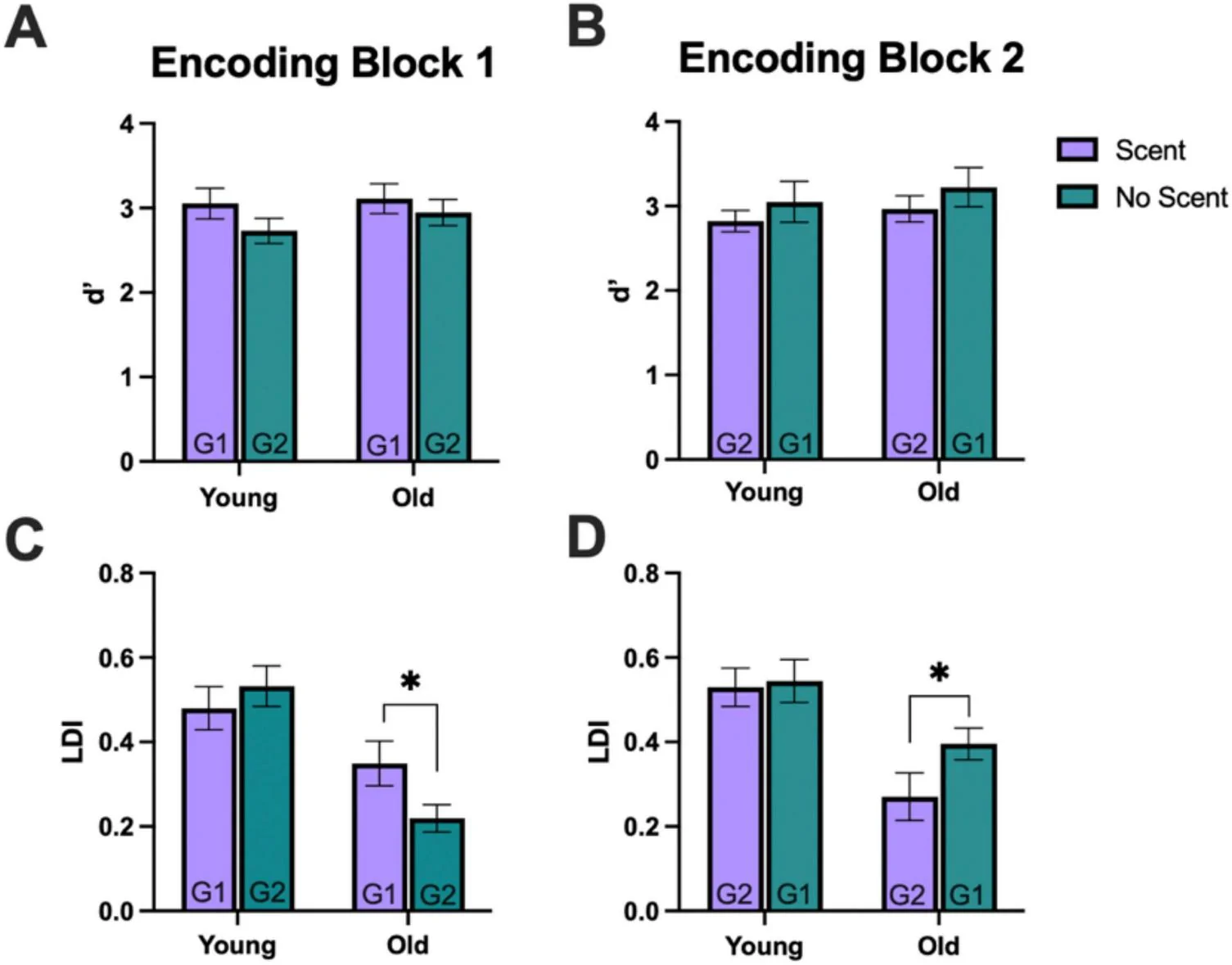
Memory performance in young and older adults across scent and order conditions. **(A,B)** Target recognition (d’) and **(C,D)** lure discrimination (LDI) in Group 1 (odor during Encoding Block 1) and Group 2 (odor during Encoding Block 2). Error bars represent standard error of the mean (SEM). *Indicates significant interaction between scent condition and order.

For lure discrimination, there was a significant scent condition × order interaction, [*F*(1,43) = 5.246, p = 0.027, η_*p*_^2^ = 0.109; Figures 3C, D]. Participants who encountered the odor during Encoding Block 1 exhibited better mnemonic discrimination for items encoded in that block compared with participants who first encoded items in the no-odor condition (Figure 3C). Conversely, participants who encountered the odor during Encoding Block 2 exhibited poorer mnemonic discrimination for those items relative to participants who encoded the same block in the no-odor condition (Figure 3D). This interaction remained significant even when controlling for odor pleasantness ratings and olfactory dysfunction measured via the UPSIT. There was also a significant effect of age [*F*(1,43) = 22.842, *p* < 0.001, η_*p*_^2^ = 0.347], where YA showed better lure discrimination than OA. No other main effects or interactions were significant (*p*’s > 0.05). When we performed a repeated-measures ANOVA within each age group, we found that order effects were primarily driven by the OA group, with a significant effect of scent condition order [*F*(1,21) = 4.628, *p* = 0.043, η_*p*_^2^ = 0.181], where OA who received the scent first performed better compared to those who received the scent second. This was not the case for YA (*p*’s > 0.05).

We also conducted a mixed-design ANOVA across scent condition (scent, no scent) and lure similarity bin (L1-L5) as within-subject factors and age group (YA, OA) and scent condition order (G1 scent first, G2 scent second) as between-subject factors to examine memory for object stimuli with varied mnemonic similarity from very high similarity (L1) to very low similarity (L5). There were no additional effects or interactions with scent condition order and similarity (*p*’s > 0.05).

### 3.3 Older adults exhibit reduced olfactory function and age-related differences account for associations between olfactory function and mnemonic discrimination

Next, we examined general olfactory dysfunction as measured via the UPSIT. The analysis of accuracy for each scent within the UPSIT across age groups is detailed in order of percent of the sample who correctly identified the odor (Figure 4A and Supplementary Table 3), in which identifiability was highest, on average, for onion (98%) and smoke (96%) and lowest for lemon (28%) and lime (34%). As expected, we found that OA showed lower scores on the UPSIT compared to young adults [*t*(45) = 2.41, *p* = 0.020, Cohen’s *d* = 0.704; Figure 4B), reflecting greater olfactory dysfunction in aging. Five OA were categorized as having severe microsmia (scores between 19 and 25), or reduced sense of smell, with seven (3 OA, 4 YA) having moderate microsmia (scores between 26 and 30), and the remaining 35 participants having mild to no microsmia (31+) (Doty et al., 1984).

**FIGURE 4:**
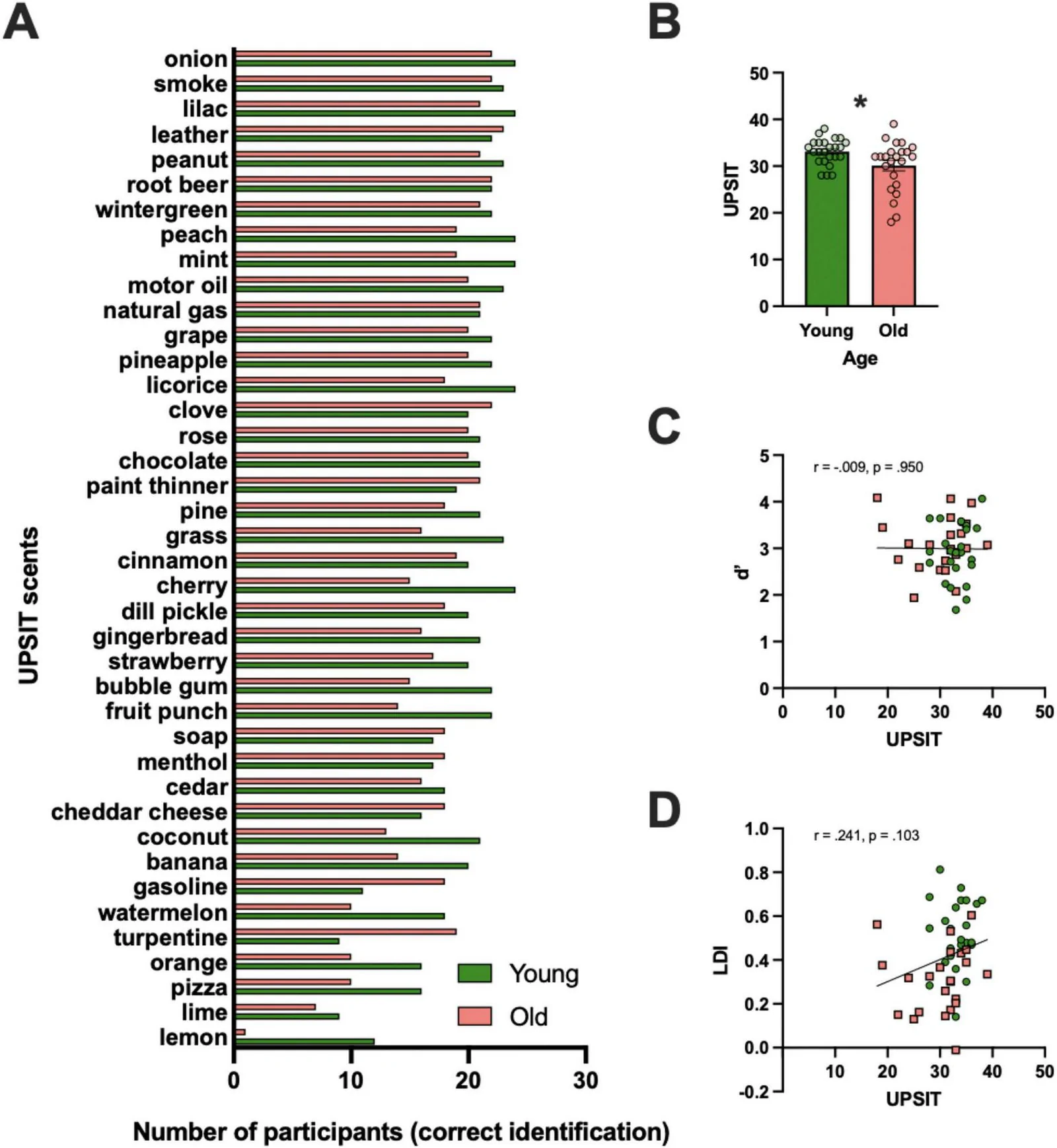
Olfactory function measured via the UPSIT across age and with memory performance. **(A)** Number of young (green) and older (red) participants who correctly identified each scent within the University of Pennsylvania Smell Identification Test (UPSIT), **(B)** difference in overall UPSIT scores across young and older adults, **(C)** correlation between UPSIT scores and target recognition (d’), **(D)** correlation between UPSIT scores and lure discrimination index (LDI). *Indicates significant overall group differences (young vs. old).

We next examined the relationship between UPSIT scores and memory performance. Overall, the UPSIT was not correlated with either d’ or LDI within each age group or across both age groups combined (*p*’s > 0.05; Figures 4C, D); however, there was a stronger relationship between UPSIT scores and LDI compared to d’ (Fisher’s r to z transform *z* = −1.721, *p* = 0.043), in which greater olfactory dysfunction was associated with worse LDI, suggesting LDI is more sensitive to olfactory dysfunction relative to target recognition. Given significant age-related differences in both olfactory function and mnemonic discrimination, we conducted an exploratory mediation analysis to examine whether age contributed to the relationship between UPSIT performance and LDI. Although UPSIT performance was not directly associated with LDI when controlling for age [β = 0.002, *t* = 0.431, *p* = 0.669] or not [β = 0.010, *t* = 1.666, *p* = 0.103], poorer UPSIT performance was associated with older age [β = −1.610, *t* = −2.276, *p* = 0.028], which in turn was associated with lower mnemonic discrimination performance [β = −0.005, *t* = −4.538, *p* < 0.001]. A significant indirect effect was observed, in which the bootstrapped unstandardized indirect effect was 0.008 and significantly differed from zero, as revealed by a 95% bootstrap confidence interval that was entirely above zero (confidence interval: 0.002–0.015), suggesting that age-related differences contributed to the observed indirect association between olfactory identification ability and mnemonic discrimination.

### 3.4 Greater olfactory dysfunction associated with reduced odor familiarity, odor pleasantness, ease of describing, and reported odor complexity

We also asked participants about the odor used in the study (lavender). We did not tell participants what the scent was and asked them to respond to a variety of questions about features of the odor, including odor familiarity, intensity, pleasantness, irritation, complexity, frequency of encounter, ease of description, and ease of imagining another time the odor was experienced. Higher UPSIT scores were associated with greater odor pleasantness (*r* = 0.445, *p* = 0.002), odor familiarity (*r* = 0.293, *p* = 0.046), ease in describing the odor (*r* = 0.413, *p* = 0.004), and odor complexity (marginally *r* = 0.270, *p* = 0.066). Exploratory moderation analyses examined whether subjective odor characteristics influenced associations between olfactory function and memory performance while controlling for age and presentation order. Among the odor features examined, only odor pleasantness significantly moderated the relationship between UPSIT and LDI. Specifically, the association between UPSIT performance and mnemonic discrimination varied across levels of perceived odor pleasantness [*b* = −0.079, *t* = −3.167, *p* = 0.003], with the strongest effects observed among individuals who rated the odor as either highly pleasant or highly unpleasant [*b* = 0.011, *t* = 3.192, *p* = 0.003]. Johnson-Neyman analyses revealed that individuals who found the odor either very pleasant or unpleasant (rating of < 5.8 or >8.3, *p* < 0.05) drove the moderation of pleasantness on the relationship between olfactory dysfunction and LDI. This was true for LDI, regardless of odor condition. We found a similar pattern for d’, but no such relationships with any of the other odor features, suggesting that odor pleasantness may influence the relationship between olfactory function and memory performance.

### 3.5 Strongest predictors of memory during odor and no odor conditions

Exploratory stepwise regression analyses were conducted to identify variables associated with memory performance during odor and no-odor encoding conditions. We included UPSIT, age, sex, education, presentation order, and all the odor features discussed above into the regression models. For LDI during the odor context, we found that age (R^2^_*adj*_ = 0.247, *b* = −0.005, *p* < 0.001) and education (R^2^_*adj*_ = 0.313, *b* = 0.027, *p* = 0.025) were the strongest predictors of LDI, predicting 32% of the variance. During the no odor context, age (R^2^_*adj*_ = 0.372, *b* = −0.005, *p* < 0.001), odor intensity (R^2^_*adj*_ = 0.427, *b* = −0.028, *p* = 0.026), and odor familiarity (R^2^_*adj*_ = 0.466, *b* = 0.027, *p* = 0.046) were the strongest predictors of LDI, predicting 47% of the variance. For d’, odor familiarity was the only predictor of memory performance during both the odor (R^2^_*adj*_ = 0.097, *b* = 0.120, *p* = 0.019) and no odor (R^2^_*adj*_ = 0.069, *b* = 0.132, *p* = 0.041) contexts, predicting only 10% and 7% of the variance, respectively.

### 3.6 Correct identification of experimental odor associated with greater familiarity and pleasantness

After the memory test, we told participants that there was a scent being diffused in one of the rooms and participants were asked to indicate what scent they smelled during the experiment (free recall), and to then rate the odor on the various odor features discussed above. Only 8 participants correctly identified lavender as the odor in the room (Correct Scent, *N* = 8, 7 YA and 1 OA), while 31 could not quite identify the specific scent, but came up with related or similar scents (e.g., floral, soap, linen) (Related Scent, *N* = 31, 15 YA and 16 OA), and 8 participants were unable to identify the odor or reported not smelling anything (Unsure, *N* = 8, 2 YA, and 6 OA). We then examined differences in odor feature ratings across the identification groups. There were significant group differences for odor familiarity [*F*(2,46) = 5.68, *p* = 0.006, η_*p*_^2^ = 0.205], odor pleasantness [*F*(2,46) = 7.56, *p* = 0.002, η_*p*_^2^ = 0.256], odor irritation [*F*(2,46) = 4.06, *p* = 0.024, η_*p*_^2^ = 0.156], and ease of imagining another time they have experienced this odor [*F*(2,46) = 3.56, *p* = 0.037, η_*p*_^2^ = 0.139]. For odor familiarity and ease of imagining a time they have experienced the odor, the correct and related groups showed higher odor familiarity compared to the unsure group. For odor pleasantness, ratings followed a linear relationship: participants who correctly identified the scent rated it as most pleasant, those who identified a related scent gave intermediate ratings, and those who were unsure rated it as least pleasant. The opposite linear relationship was demonstrated for odor irritation: participants who correctly identified the scent rated it as least irritating, those who identified a related scent gave intermediate ratings, and those who were unsure rated it as most irritating.

## 4 Discussion

The present study examined whether odor context during encoding influences hippocampal-dependent memory in young and older adults. Contrary to our primary hypothesis, the presence of a lavender odor during encoding did not improve target recognition or mnemonic discrimination overall. However, several findings emerged that provide insight into the relationship between olfaction and memory across aging. First, odor context influenced mnemonic discrimination in older adults depending on when the odor was encountered during learning. Second, older adults exhibited expected declines in both olfactory function and mnemonic discrimination. Finally, individual differences in odor perception, particularly odor pleasantness and familiarity, were associated with variability in memory performance. Together, these findings suggest that odor context does not uniformly enhance memory, but may influence hippocampal-dependent memory under specific conditions related to age, timing of exposure, and subjective odor experience.

### 4.1 Odor context does not uniformly enhance memory performance

The absence of a main effect of odor context on either target recognition or mnemonic discrimination suggests that the mere presence of a generally pleasant odor during encoding may not be sufficient to enhance memory. These findings are consistent with previous work indicating that the effects of odor on memory depend on multiple factors, including the salience of the odor, attentional engagement, emotional significance, and congruency between the odor and the encoded material (Herz, 2016; Rouby et al., 2009). Although odor enrichment interventions have demonstrated cognitive benefits in older adults (Woo et al., 2023), our findings suggest that simply introducing an odor during encoding does not automatically improve memory performance.

Importantly, odor context also failed to influence target recognition, suggesting that any potential effects of odor may not operate at the level of general recognition memory. Instead, odor-related influences may be more likely to emerge for hippocampal-dependent memory processes that require discrimination among overlapping experiences. Future studies should investigate whether more salient, emotionally arousing, or personally meaningful odors produce stronger effects on mnemonic discrimination than the relatively familiar and low-arousal lavender odor used in the present study.

### 4.2 Timing of odor exposure influences mnemonic discrimination in older adults

The most notable finding was that odor presentation order influenced mnemonic discrimination in older adults. Older adults who encountered the odor context during the first encoding block exhibited better lure discrimination than those who encountered the odor during the second encoding block. This effect was selective to mnemonic discrimination and did not extend to target recognition. Notably, odor presentation order influenced mnemonic discrimination but not target recognition, suggesting that contextual odor cues may preferentially affect hippocampal-dependent discrimination processes rather than general recognition memory. Because mnemonic discrimination is thought to rely on hippocampal pattern separation, these behavioral findings are consistent with the possibility that odor context influences hippocampal-dependent discrimination; however, the present study cannot directly establish a neural mechanism. One possible explanation is that odor context served as a salient environmental feature that may have facilitated item-context binding when present at the outset of learning. According to contextual binding accounts of aging, older adults often experience difficulty integrating items with their surrounding contextual features, resulting in reduced representational specificity and increased interference at retrieval (Dulas and Duarte, 2011; Gutchess et al., 2007; Chee et al., 2006). Introducing an odor context early in encoding may therefore provide a distinctive contextual framework that supports the formation of more differentiated memory representations and reduces interference among similar experiences.

A related explanation comes from encoding specificity and context-dependent memory theories, which propose that contextual features encoded alongside an experience can later serve as effective retrieval cues. Consistent with encoding specificity and context-dependent memory theories, contextual cues may provide the greatest benefit when they are incorporated into the memory trace during initial encoding (Tulving and Thomson, 1973; Godden and Baddeley, 1975). In contrast, introducing a novel contextual cue after learning has already begun may provide less opportunity for the cue to become integrated with subsequently encoded information. This possibility is consistent with evidence that older adults may be less flexible in adapting to changes in environmental context during learning (Kimura et al., 2019). Thus, odor context may function less as a general mnemonic enhancer and more as an encoding scaffold that is most beneficial when present early in the learning process. This interpretation is further supported by evidence that odors can influence neural activity during memory formation. Odor presentation during encoding has been shown to elicit sustained neural activity in memory-related brain regions, suggesting that olfactory cues may become integrated into the neural representation of an experience and subsequently contribute to retrieval processes (Tarrida et al., 2025). Such findings raise the possibility that odor context may influence mnemonic discrimination not only by serving as an external retrieval cue, but also by shaping the encoding environment in ways that promote the formation of more distinctive memory representations. Importantly, these effects were observed only in older adults. Rather than reflecting ceiling performance in younger adults, the absence of odor-related benefits may indicate that younger adults rely less on external contextual support because mnemonic discrimination processes remain relatively intact. Older adults, in contrast, may derive greater benefit from environmental cues that support the formation or retrieval of distinctive memory representations.

### 4.3 Aging is associated with declines in both olfactory function and mnemonic discrimination

Consistent with previous work, older adults demonstrated poorer mnemonic discrimination and reduced olfactory identification ability relative to younger adults. These findings align with evidence that aging affects both hippocampal-dependent memory processes and olfactory functioning (Doty and Kamath, 2014; Holden et al., 2013; Stark et al., 2013). Although olfactory identification performance was not directly associated with mnemonic discrimination, exploratory analyses suggested that age contributed substantially to variance in both olfactory function and mnemonic discrimination. These findings suggest that declines in olfactory processing and hippocampal-dependent memory may emerge in parallel across aging, even if they are not tightly coupled at the individual level. Given the close anatomical and functional relationships between olfactory pathways and medial temporal lobe structures (Sullivan et al., 2015; Zhou et al., 2021), future studies using larger samples and more sensitive measures of olfactory processing may help determine whether these age-related declines reflect shared underlying mechanisms.

### 4.4 Individual differences in odor perception shape memory-related effects

The present findings also highlight the importance of individual differences in odor perception. Participants with better olfactory identification ability reported greater familiarity with the odor, greater pleasantness, and greater ease describing the odor. Furthermore, exploratory analyses indicated that subjective pleasantness ratings influenced the relationship between olfactory function and mnemonic discrimination. These findings are consistent with prior work demonstrating that odor perception varies substantially across individuals and that factors such as familiarity, emotional valence, and personal relevance can influence cognitive responses to odors (Herz, 2016; Rouby et al., 2009). Because odors are strongly connected to limbic and memory-related brain regions, subjective evaluations of an odor may alter the degree to which the odor is encoded as part of an episodic memory trace. Exploratory regression analyses further emphasized the importance of these individual differences. Although age was the strongest predictor of mnemonic discrimination, odor familiarity emerged as a modest predictor of memory performance across conditions. Together, these findings suggest that variability in odor perception may contribute to variability in the effectiveness of odor context as a memory cue.

### 4.5 Odor identification and awareness

Few participants correctly identified lavender as the experimental odor, although most participants reported smelling something and many generated semantically related descriptors such as “floral,” “soap,” or “linen.” Participants who correctly identified the odor reported greater familiarity and pleasantness, suggesting that subjective experience of the odor influenced the likelihood of successful identification. The relatively low rate of explicit odor identification raises interesting questions regarding the role of conscious awareness in odor-related memory effects. Some studies suggest that conscious recognition enhances the ability of odors to serve as retrieval cues (Herz and Schooler, 2002), whereas others demonstrate that odors can influence cognition and behavior even when they are not consciously recognized (Li et al., 2007; Epple and Herz, 1999). The present findings suggest that odor familiarity and pleasantness may be particularly important factors determining whether an odor becomes integrated into a memory representation, regardless of whether the odor is explicitly identified.

### 4.6 Limitations and future directions

Several limitations should be considered. First, odor presentation occurred through a passive diffuser and may not have produced a sufficiently salient olfactory experience to maximize contextual effects. Future studies should systematically manipulate odor intensity, novelty, emotional valence, and personal relevance to determine which characteristics most effectively influence mnemonic discrimination. Second, odor and no-odor encoding blocks were conducted in separate testing rooms to minimize odor contamination. Although this design reduced carryover effects, it introduced a change in physical context that may also have influenced encoding. Future studies should evaluate alternative approaches for odor administration that maintain consistent spatial context while preventing residual odor contamination. Third, the relatively small sample size limited statistical power for detecting modest associations between olfactory function and memory performance and constrained interpretation of several exploratory analyses. Replication in larger samples and longitudinal designs will be important for establishing the reliability of the observed effects. Similarly, although sensitivity analyses indicated that inclusion of sex, race, or education did not alter the conclusions regarding the primary age-related effects, the unequal distribution of sex and race across age groups and the small sample size limited our ability to evaluate these effects thoroughly and warrant consideration in future studies with larger, more balanced samples. Finally, future work should further examine how olfactory context interacts with hippocampal computations across aging, ideally incorporating neuroimaging approaches capable of assessing hippocampal and olfactory network function directly. Understanding how olfactory processing influences memory may ultimately inform interventions designed to support cognitive health during aging.

### 4.7 Conclusion

Odor context did not enhance memory performance overall; however, the effects of odor depended on age, timing of exposure, and individual differences in odor perception. Older adults exhibited improved mnemonic discrimination when odor context was encountered early during learning, suggesting that odor cues may support memory under specific encoding conditions. Together, these findings provide new evidence that olfactory influences on memory are highly context dependent and highlight the importance of considering both aging and individual differences when evaluating odor-based approaches to support memory function.

## Data Availability

The datasets presented in this study can be found in online repositories. The names of the repository/repositories and accession number(s) can be found in the article/Supplementary material.
