# Supplementary material for: Olfactory context and memory across the adult lifespan: effects on mnemonic discrimination and olfactory function
Source: Front Aging Neurosci. 2026 Sep 28;18:1917760. doi: 10.3389/fnagi.2026.1917760 (PMC13656307; doi:10.3389/fnagi.2026.1917760)
Supplement: Supplementary file 1 [file Data_Sheet_1.docx]

**Supplementary Information 1**

**Odor Room:**

- Diffuser → Participant: 75in / 190.5cm
- Eyes → Screen: 26in / 66cm
- Room Length: 195in / 496cm
- Room Width: 119.5in / 304cm
- Room Height: 114.5in / 291cm

**Non-Odor Room:**

| **Supplementary Table 1. Number of object stimuli across similarity** | | | |
| --- | --- | --- | --- |
| **Variable** | **Encoding Block 1** | **Encoding Block 2** | **Retrieval** |
| N | 64 | 64 | 192 |
| Foils | 0 | 0 | 64 |
| Targets | 32 | 32 | 64 |
| Similar Lures 1 | 7 | 6 | 13 |
| Similar Lures 2 | 6 | 7 | 13 |
| Similar Lures 3 | 7 | 6 | 13 |
| Similar Lures 4 | 6 | 7 | 13 |
| Similar Lures 5 | 6 | 6 | 12 |

**Supplementary Table 2. Behavioral response by trial type**

|  |  | Behavioral response | | | |
| --- | --- | --- | --- | --- | --- |
| Scent Condition | Stimulus Type | “Same” | | “New or Different” | |
| Scent |  |  |  |  |  |
|  | Foils | 0.04 (0.01) | 0.04 (0.01) | 0.96 (0.01) | 0.96 (0.01) |
|  | Targets | 0.83 (0.02) | 0.86 (0.02) | 0.17 (0.02) | 0.14 (0.02) |
|  | Similar Lures 0 | 0.33 (0.03) | 0.55 (0.04) | 0.67 (0.03) | 0.45 (0.04) |
|  | Similar Lures 1 | 0.52 (0.04) | 0.74 (0.05) | 0.48 (0.04) | 0.26 (0.05) |
|  | Similar Lures 2 | 0.44 (0.05) | 0.71 (0.03) | 0.56 (0.05) | 0.29 (0.03) |
|  | Similar Lures 3 | 0.29 (0.05) | 0.53 (0.04) | 0.71 (0.05) | 0.47 (0.04) |
|  | Similar Lures 4 | 0.25 (0.03) | 0.43 (0.05) | 0.75 (0.03) | 0.57 (0.05) |
|  | Similar Lures 5 | 0.06 (0.01) | 0.11 (0.02) | 0.94 (0.01) | 0.89 (0.02) |
| No Scent |  |  |  |  |  |
|  | Foils | 0.04 (0.01) | 0.04 (0.01) | 0.96 (0.01) | 0.96 (0.01) |
|  | Targets | 0.82 (0.02) | 0.86 (0.02) | 0.18 (0.02) | 0.14 (0.02) |
|  | Similar Lures 0 | 0.28 (0.02) | 0.55 (0.03) | 0.72 (0.02) | 0.45 (0.03) |
|  | Similar Lures 1 | 0.46 (0.04) | 0.75 (0.03) | 0.54 (0.04) | 0.25 (0.03) |
|  | Similar Lures 2 | 0.34 (0.04) | 0.66 (0.05) | 0.66 (0.04) | 0.34 (0.05) |
|  | Similar Lures 3 | 0.23 (0.04) | 0.52 (0.04) | 0.77 (0.04) | 0.48 (0.04) |
|  | Similar Lures 4 | 0.21 (0.03) | 0.45 (0.04) | 0.79 (0.03) | 0.55 (0.04) |
|  | Similar Lures 5 | 0.06 (0.01) | 0.13 (0.02) | 0.94 (0.01) | 0.87 (0.02) |

*Mean, SEM; Similar Lures 0 = total number of similar lures across all similarity bins

| **Supplementary Table 3. UPSIT odor item identification rates** | | | |
| --- | --- | --- | --- |
| **Variable, n (%)** | **Total (n = 47)** | **Young (n = 24)** | **Old (n = 23)** |
| Pizza | 26 (55.32) | 16 (66.67) | 10 (43.48) |
| Bubble gum | 37 (78.72) | 22 (91.67) | 15 (65.22) |
| Menthol | 35 (74.47) | 17 (70.83) | 18 (78.26) |
| Cherry | 39 (82.98) | 24 (100.00) | 15 (65.22) |
| Motor oil | 43 (91.49) | 23 (95.83) | 20 (86.96) |
| Mint | 43 (91.49) | 24 (100.00) | 19 (82.61) |
| Banana | 34 (72.34) | 20 (83.33) | 14 (60.87) |
| Clove | 42 (89.36) | 20 (83.33) | 22 (95.65) |
| Leather | 45 (95.74) | 22 (91.67) | 23 (100.00) |
| Coconut | 34 (72.34) | 21 (87.50) | 13 (56.52) |
| Onion | 46 (97.87) | 24 (100.00) | 22 (95.65) |
| Fruit punch | 36 (76.60) | 22 (91.67) | 14 (60.87) |
| Licorice | 42 (89.36) | 24 (100.00) | 18 (78.26) |
| Cheddar cheese | 34 (72.34) | 16 (66.67) | 18 (78.26) |
| Cinnamon | 39 (82.98) | 20 (83.33) | 19 (82.61) |
| Gasoline | 29 (61.70) | 11 (45.83) | 18 (78.26) |
| Strawberry | 37 (78.72) | 20 (83.33) | 17 (73.91) |
| Cedar | 34 (72.34) | 18 (75.00) | 16 (69.57) |
| Chocolate | 41 (87.23) | 21 (87.50) | 20 (86.96) |
| Gingerbread | 37 (78.72) | 21 (87.50) | 16 (69.57) |
| Lilac | 45 (95.74) | 24 (100.00) | 21 (91.30) |
| Turpentine | 28 (59.57) | 9 (37.50) | 19 (82.61) |
| Peach | 43 (91.49) | 24 (100.00) | 19 (82.61) |
| Root beer | 44 (93.62) | 22 (91.67) | 22 (95.65) |
| Dill pickle | 38 (80.85) | 29 (83.33) | 18 (78.26) |
| Pineapple | 42 (89.36) | 22 (91.67) | 20 (86.96) |
| Lime | 16 (34.04) | 9 (37.50) | 7 (30.43) |
| Orange | 26 (55.32) | 16 (66.67) | 10 (43.48) |
| Wintergreen | 43 (91.49) | 22 (91.67) | 21 (91.30) |
| Watermelon | 28 (59.57) | 18 (75.00) | 10 (43.48) |
| Paint thinner | 40 (85.11) | 19 (79.17) | 21 (91.30) |
| Grass | 39 (82.98) | 23 (95.83) | 16 (69.57) |
| Smoke | 45 (95.74) | 23 (95.83) | 22 (95.65) |
| Pine | 39 (82.98) | 21 (87.50) | 18 (78.26) |
| Grape | 42 (89.36) | 22 (91.67) | 20 (86.96) |
| Lemon | 13 (27.66) | 12 (50.00) | 1 (4.35) |
| Soap | 35 (74.47) | 17 (70.83) | 18 (78.26) |
| Natural gas | 42 (89.36) | 21 (87.50) | 21 (91.30) |
| Rose | 41 (87.23) | 21 (87.50) | 20 (86.96) |
| Peanut | 44 (93.62) | 23 (95.83) | 21 (91.30) |
